# 669. Comparison of Actual versus Predicted Sweat Rates During Physical Activity in Well-healed Burn Survivors

**DOI:** 10.1093/jbcr/irag033.550

**Published:** 2026-04-06

**Authors:** Craig G Crandall, Timothy Schaefer, Elizabeth Gideon, Zachary McKenna, Ollie Jay, Jacob L McNary, Rachel Cottle

**Affiliations:** University of Texas Southwestern Medical Center, Dallas, TX; Texas Health Presbyterian Hospital Dallas, Dallas, TX; University of Texas Southwestern Medical Center, Dallas, TX; University of Arkansas, Fayetteville, AR; University of Sydney, Sydney, NSW; Texas Health Presbyterian Hospital Dallas, Dallas, TX; University of Texas Southwestern Medical Center, Dallas, TX

## Abstract

**Introduction:**

Sweating is critical for human thermoregulation, particularly in warm environmental conditions. A free online tool (sweatratecalculator.com) provides guidance on fluid intake needs during physical activity based on predicted whole-body sweat rates of non-burned injured individuals. The accuracy of this sweat rate prediction tool has not been assessed in burn survivors. Given that grafted skin does not sweat, this tool may over-predict actual sweat rates in burn survivors during physical activity. Conversely, heightened core temperatures during physical activity in burn survivors may result in greater sweating from non-injured areas, perhaps culminating in similar whole-body sweat rates relative to non-burned injured individuals. This study tested the former hypothesis that the sweat rate prediction tool will over-predict actual sweat loss in burn survivors following physical activity.

**Methods:**

In 80 burn survivors (17 to 84% body surface area burned), whole-body sweat rates were measured from changes in nude body mass, after accounting for fluid intake and urine output, following 40 - 90 min of mild/moderate intensity (4-5 METS) physical activity in thermoneutral and warm environmental conditions. The measured sweat rates were compared with predicted sweat rates obtained from the aforementioned online tool.

**Results:**

The Bland–Altman plot shows a proportional bias (i.e., a negative slope, *p*<.001; see Figure), resulting in the tool over predicting measured sweat rates by an average of 0.19 ± 0.22 l/hr in burn survivors having sweat rates less than 0.4 l/hr (i.e., lower whole-body sweat rates), while under predicting measured sweat rates by an average of 0.14 ± 0.17 l/hr in burn survivors having sweat rates greater than 0.9 l/hr (i.e., higher whole-body sweat rates). The percent body surface area burned did not influence this proportional bias.

**Conclusions:**

The online sweat rate prediction tool may be useful to estimate whole-body sweat rates in burn survivors, while recognizing the potential to over-estimate actual sweat rates in those with low sweating responses and under-estimate actual sweat rates in those with high sweating responses.

**Applicability of Research to Practice:**

These findings inform the burn survivor and rehabilitation practitioner that fluid intake needs of burn survivors during physical activity can be generally estimated using the online sweat rate prediction tool, recognizing the potential error for burn survivors with low and high sweating rates.

**Funding for the study:**

NIH-National Institutes of General Medical Sciences.

